# Challenging Host Specificity: Malacosporean Parasite Dynamics—*Tetracapsuloides bryosalmonae* Beyond Salmonids—An Infection Experiment

**DOI:** 10.1111/jfd.70233

**Published:** 2026-06-28

**Authors:** Céline Möckli, Nicolas Diserens, Gary Delalay, Gastón Moré, Heike Schmidt‐Posthaus

**Affiliations:** ^1^ Institute for Fish and Wildlife Health, Department of Infectious Diseases and Pathobiology, Vetsuisse Faculty University of Bern Bern Switzerland; ^2^ Institute of Parasitology, Department of Infectious Diseases and Pathobiology, Vetsuisse Faculty University of Bern Bern Switzerland

**Keywords:** brown trout, eDNA, experimental infection, minnow, pathology, Proliferative Kidney Disease

## Abstract

Proliferative Kidney Disease (PKD) is a severe parasitic disease of salmonids caused by the malacosporean endoparasite *Tetracapsuloides bryosalmonae*. In the fish intermediate host, the parasite is disseminated via the circulatory system and ultimately reaches the kidney, where it matures before releasing infective spores into the environment through urine. Disease severity in salmonids is strongly temperature dependent, with prolonged exposure to temperatures above 15°C promoting parasite proliferation and pathology. We hypothesised that non‐salmonid fish species better adapted to warmer conditions may serve as additional intermediate or reservoir hosts in the parasite life cycle. In a controlled laboratory infection experiment, minnow ( *Phoxinus phoxinus* ) were exposed to infectious malacospores released from *T. bryosalmonae*‐infected bryozoans, the final host in the parasite cycle. Infection was confirmed by Sanger and Nanopore deep amplicon sequencing, and spore excretion in urine was demonstrated by eDNA. Notably, minnow showed no renal pathology and no mortality. These findings expand the recognised host range of *T. bryosalmonae* and suggest that minnow may contribute to parasite persistence in river systems, particularly under warming conditions that extend the transmission season and increase infection pressure.

## 1 Introduction

Proliferative Kidney Disease (PKD) is a severe parasitic disease of salmonid fish caused by the malacosporean endoparasite *Tetracapsuloides bryosalmonae* (El‐Matbouli et al. 1992; Hedrick et al. 1993; Okamura et al. 2011). There are two malacosporean genera, *Tetracapsuloides* and *Buddenbrockia* (Canning et al. 2000; Naldoni et al. 2019). *T. bryosalmonae* has a complex diheteroxenous life cycle alternating between freshwater bryozoans (*Phylactolaemata*) as final hosts and fish, for which only salmonids are currently recognized, as intermediate hosts (Anderson et al. 1999; Feist and Bucke 1993; Hedrick et al. 1993; Longshaw et al. 1999; Okamura et al. 2001; Sudhagar et al. 2019). Infection of fish occurs when bryozoan‐derived malacospores penetrate the gills and enter the circulatory system, disseminating to internal organs and ultimately the kidney (Grabner and El‐Matbouli 2010b; Morris et al. 2002). Within the renal tissue, the parasite proliferates as extrasporogonic stages before differentiating into sporogonic forms in the tubular lumen (Kent and Hedrick 1985), which then release infective spores into the environment through the urine, completing the life cycle (Bettge, Segner, et al. 2009; Bettge, Wahli, et al. 2009; Hedrick et al. 2004; Morris et al. 2002). Therefore, infectious life stages of *T. bryosalmonae* can only be released in a substantial manner by living hosts. In case of severe PKD and intermediate host's death, vastly immature parasite live stages or non‐infectious spores reach the water.

PKD has been documented in several salmonid species, including brown trout (*Salmo trutta fario*), brook trout ( *Salvelinus fontinalis* ), Atlantic salmon ( *Salmo salar* ), grayling ( *Thymallus thymallus* ), and rainbow trout ( *Oncorhynchus mykiss* ), with marked interspecific variation in susceptibility and disease severity (Bettge, Wahli, et al. 2009; Mo et al. 2011; Schmidt‐Posthaus et al. 2012; Schmidt‐Posthaus et al. 2017; Sterud et al. 2007; Vasemägi et al. 2017). Young‐of‐the‐year (YOY) salmonids are particularly vulnerable, often developing pronounced granulomatous nephritis with widespread necrosis, haemorrhage, and thrombi formation (Bettge, Wahli, et al. 2009; Hedrick et al. 1993). These renal lesions likely contribute to increased mortality by impairing erythropoiesis and immune function, thereby modulating thermal tolerance and metabolic rate as well as increasing susceptibility to secondary infections (Bruneaux et al. 2017; Gorgoglione et al. 2020; Hedrick et al. 1993; Schmidt‐Posthaus et al. 2013).

Disease progression is strongly temperature‐dependent, with extended periods above 15°C promoting parasite proliferation and pathology in the fish host (Bettge, Wahli, et al. 2009; Okamura et al. 2011; Rubin et al. 2019; Wahli et al. 2008). Consequently, PKD outbreaks display strong seasonality in the Northern Hemisphere and nowadays impose substantial ecological impacts on wild salmonids as well as significant economic losses to aquaculture (Clifton‐Hadley et al. 1986; Ferguson and Ball 1979; Naas et al. 2023). The life cycle of *T. bryosalmonae* in salmonids likely reflects long‐term adaptation to salmonid hosts inhabiting cold, well‐oxygenated waters. Historically, infections with *T. bryosalmonae* probably caused little to no disease, as commonly observed for many myxozoan parasites (Hartikainen et al. 2016). Data from recent disease outbreaks observed in northern regions suggest indeed that *T. bryosalmonae* infections have long been widespread but remained inconspicuous until environmental changes, such as rising water temperatures because of climate change, environmental degradation (e.g., eutrophication), and altered host–parasite dynamics (El‐Matbouli and Hoffmann 2002; Kristmundsson et al. 2023).

Non‐salmonid fish species may serve as alternative intermediate hosts or reservoir hosts within the parasite life cycle, thereby maintaining the environmental contamination with infective *T. bryosalmonae* spores. Preliminary screening of wild fish co‐occurring with infected brown trout indicated that several non‐salmonid species may harbour *T. bryosalmonae* DNA (Stelzer et al. 2024, unpublished PhD thesis). Among these potential non‐salmonid hosts, minnow ( *Phoxinus phoxinus* ) (Family: Leuciscidae) is especially noteworthy because they have historically been widely used as live‐bait in recreational fisheries, placing them at the centre of both ecological and management concerns (Gozlan et al. 2010; Scharnweber 2020). In recent years, however, live‐bait use has been restricted or banned in some countries, including Switzerland (Animal Protection Ordinance 2008), so their relevance varies across both space and time.

Recent studies have substantially broadened current understanding of malacosporean diversity and, importantly, indicate that these parasites are more generalists than previously assumed. New species described by Bartošová‐Sojková et al. (2014) and Hartikainen et al. (2014), along with the multi‐host detections reported by Patra et al. (2017), demonstrate that *Tetracapsuloides* species occur across a wide array of hosts. Patra et al. (2017) identified five *Tetracapsuloides* species in both non‐salmonid fish (*Tetracapsuloides* spp. 2–5) and bryozoans (*Tetracapsuloides* sp. 1). Their experimental transmission study further showed that *Tetracapsuloides vermiformis* (formerly *Tetracapsuloides* sp. 3) can cycle between the bryozoan *Fredericella sultana* and common carp ( *Cyprinus carpio* ), confirming the role of carp as an intermediate host and underscoring flexible host associations of the genus *Tetracapsuloides*. Additional molecular evidence reinforces the notion that malacosporeans exploit a broad host spectrum. Naldoni et al. (2019) detected *Tetracapsuloides* spp. in five different fish species through PCR and sequencing, supported by ultrastructural analyses. Their findings showed *T. bryosalmonae* infecting brown trout, rainbow trout, and whitefish ( *Coregonus albula* ), while *Tetracapsuloides* sp. 4 occurred in roach ( *Rutilus rutilus* ) and *Tetracapsuloides* sp. 5 in gudgeon ( *Gobio gobio* ). Although *T. bryosalmonae* is still the only malacosporean consistently reported from salmonid tissues, the growing number of species detected in diverse fish hosts indicates that malacosporeans, rather than being strict specialists, exhibit a considerable degree of host generalism.

Expanding the recognized host range of *T. bryosalmonae* would fundamentally reshape our understanding of PKD ecology and persistence in freshwater ecosystems. Climate projections predicting increasingly longer and more frequent warm‐water periods are expected to extend the seasonal window of parasite transmission and elevate infection pressure (Okamura et al. 2011; Ros et al. 2022; Rubin et al. 2022). If warm‐water non‐salmonid fish species, such as minnow, prove to be competent hosts, they could facilitate parasite persistence under conditions increasingly unsuitable for salmonids. Such alternative hosts could therefore intensify long‐term environmental parasite burden and long‐term infection pressure on salmonids, potentially reducing their range of distribution (Rubin et al. 2022).

To address these knowledge gaps, we conducted a laboratory infection experiment in which minnow were exposed to infectious malacospores released from *T. bryosalmonae*‐infected bryozoans under controlled conditions. This experimental approach was designed to directly test host competence by determining whether minnow can become infected, support parasite development, including excretion of spores, and develop characteristic pathological changes. Specifically, we aimed to (i) evaluate the susceptibility of minnow to *T. bryosalmonae* infection and (ii) characterize associated pathology, mortality, and excretion capability under experimental conditions.

## 2 Material and Methods

### 2.1 Ethics Statement

The project followed the regulations of the Swiss Animal Welfare Act (SR 455) and Swiss Animal Welfare Ordinance (SR 455.1). All experiments were approved by the Cantonal Veterinary Office Bern, Switzerland under Authorization #BE3/2024.

### 2.2 Experimental Setup and Initial Fish Health Check

In March 2024, 158 adult minnows were obtained from a local Swiss fish trader and transported to the Institute for Fish and Wildlife Health (FIWI), University of Bern. At FIWI, the minnows were acclimatized for 56 days, after which a subset of 20 individuals underwent a full health examination, including testing for *T. bryosalmonae* DNA. An additional ten minnows were only screened for the presence of *T. bryosalmonae* DNA. The remaining minnows were allocated to three infection groups (Tanks Minnow, TM1–3) of 32 fish each and one unexposed control group (Tank Unexposed, TU), also comprising 32 fish.

Fifty YOY brown trout were obtained from a commercial fish farm, Switzerland, with no previous history of PKD. The trout were acclimatized for 28 days, after which 10 individuals underwent health screening, including testing for *T. bryosalmonae* DNA. Additional 10 animals were only tested for parasite DNA. The remaining 22 trout were used in the infection experiment (Tank Brown Trout, TB).

All aquaria consisted of 38 L glass tanks supplied with tap water under flow‐through conditions, with constant aeration and a 12:12 h light–dark cycle. Water temperature was maintained at 18°C for both species.

For the initial health check, minnow and brown trout were euthanized with an overdose of buffered tricaine (150 mg/L (brown trout) and 500 mg/L (minnow) tricaine methanesulfonate; Tricaine PHARMAQ 1000 mg/g, Pharmaq, Overhalla, Norway). A complete necropsy was performed and macroscopical changes on external and internal organs were documented. Native smears of external (skin and gills) and internal (gut) tissues were microscopically analysed for detection of parasites, and samples from spleen, liver and kidney were cultured on blood agar plates (Bio Mérieux, Marcy‐l'Étoile, Switzerland) and incubated at 22°C for 48 h for bacteriological analysis. In addition, samples of gill and spleen were cultivated on selective agar plates (Anacker and Ordal 1959) for 5 days at 15°C for cultivation of *Flavobacteriales*. The kidney was dissected longitudinally, with one half immediately frozen at −20°C for subsequent qPCR analysis to detect *T. bryosalmonae* DNA. The other half of the kidney and all remaining organs were fixed in 10% buffered formalin for histological examination. Animals that died during the experiment underwent full necropsy, followed if possible by bacteriological and parasitological examinations, as well as *T. bryosalmonae* DNA analysis as described above.

### 2.3 Parasite Exposure

On 21st September 2024, freshwater bryozoans were collected from Swiss rivers known to be endemic for the parasite (Surb, coordinates 47.532663/8.298328; Lyssbach, coordinates 47.049958/7.355854; Furtbach, coordinates 47.447748/8.376322; and Alte Aare, coordinates 47.111546/7.317167) and transferred immediately to FIWI. Two zooids from each sampling site were screened for viability and presence of infective parasite sacs under a Nikon Digital Sight 1000 stereomicroscope (Nikon Corporation, Tokio, Japan). This bryozoan zooid tissue was disrupted by grinding, and DNA was extracted from two 2 mL homogenates. A qPCR was performed to investigate presence of *F. sultana* DNA (most common bryozoan host) according to Carraro et al. (2017) and presence of *T. bryosalmonae* DNA according to Bettge, Wahli, et al. (2009), as well as to calculate approximate parasite DNA amount in bryozoa. Briefly, for each qPCR run, a standard curve was generated using gBlock concentrations (Integrated DNA Technologies Inc., Coravilla, IA, USA) of 10^−5^, 10^−6^, 10^−7^, and 10^−8^. Copy numbers are based on gBlocks information sheet with known amount of fmoles/ng, molecular weight and the amount of μl used during one qPCR reaction. The Ct values of each sample, analysed in triplicate, were used to calculate copy numbers based on the corresponding standard curve. The parasite homogenate had a concentration of 48,149 copies/μL. Prior to exposure, the water flow was stopped to avoid dilution of the spores, aeration increased, and tank water lowered to 20% (approx. 7.6 L).

Exposure of minnow and brown trout was conducted over three consecutive days. During day one and day two, overflow water of the buckets in which the bryozoa were kept (approx. 2 L) was combined and distributed evenly to TM1, TM2, TM3 and TB (approx. 500 mL/tank). The same water volume of tap water was added to the unexposed minnow (TU). During exposure, fish were monitored constantly. After 1.5 h, the water flow was restarted. The buckets in which the bryozoa were kept were refilled with designated river water. On day three, the remaining bryozoa colonies were homogenized with an immersion blender. The same amount of mixture (50 mL) was added to all exposed groups. The same amount of tap water was added to the unexposed minnow.

### 2.4 Fish Sampling and Histology

The infection experiment lasted for 120 days, and four samplings were performed on days 38–41, 69, 83, and 105 post exposure (dpe). Sampling procedures for fish (minnow and brown trout) were carried out in control and all exposed groups separately to avoid cross contamination. Five fish/tank were randomly selected and euthanized using an overdose of buffered tricaine. A complete necropsy was performed as described above. The kidney was dissected longitudinally, with one half immediately frozen at −20°C for further molecular examination and the other half fixed in 10% buffered formalin for histology. After 24 h fixation, kidney tissue was processed following standard paraffin embedding procedures. After dehydration and xylene exchange, tissues were infiltrated with paraffin wax and embedded into a wax block. The blocks were cut at 4 μm sections and stained with haematoxylin and eosin (HE). The evaluation was performed using an Eclipse E400 microscope (Nikon Corporation, Tokio, Japan). Histopathological changes associated with PKD were evaluated according to Bettge, Wahli, et al. (2009) and Schmidt‐Posthaus et al. (2012) and parasite structures were searched.

### 2.5 Water Sampling

Between days 20 and 105 pe, aquarium water was filtered once weekly, except at weeks 8, 9, and 10 pe, when it was filtered twice weekly. The increased sampling frequency was based on previous experimental findings by Strepparava et al. (2018) demonstrating the start of *T. bryosalmonae* shedding in brown trout, maintained at 15°C, from approximately 55 dpe onwards. At each sampling point, the water volume of each exposed tank (minnow and brown trout) was lowered to 65% (24 L). Water flow was stopped for 1 h, aeration was increased and continuous supervision of fish behaviour and/or macroscopic changes was performed. Afterwards, four biological filter replicates, each containing 0.6 L, corresponding to 10% of the tank water volume (in total 2.4 L) were filtered through 0.45 μm Sterivex filters (Merck Millipore, Billerica, MA, USA) and sealed (Stelzer et al. 2024). Water in unexposed control aquaria was sampled simultaneously. After sampling, aquarium water flow was re‐started. Filters were stored at −20°C until DNA extraction.

### 2.6 DNA Extraction

DNA was extracted from kidney tissue using Blood and Tissue Kit (QIAGEN N.V., Venlo, Netherlands), following the manufacturer's protocol. As negative extraction control, DNAse free water was used. DNA concentration and quality were checked with NanoDrop One UV–Vis Spectrophotometer (Thermo Fisher Scientific, Wilmington, USA). Negative controls never tested positive for presence of DNA.

Tissue from brown trout and minnow were always handled separately.

Environmental DNA (eDNA) was recovered from Sterivex filter cartridges, following the protocol of Stelzer et al. (2024). Extraction control was performed using Sterivex filters without filtering water. DNA concentration and quality were checked with NanoDrop One UV–Vis Spectrophotometer. Negative controls never tested positive for the presence of DNA.

### 2.7 qPCR, PCR and Sanger Sequencing

Kidney tissue extracts of every sampled fish were tested by probe‐based qPCR according to Bettge, Segner, et al. (2009), targeting the 73 bp amplicon of the *T. bryosalmonae* 18 small subunit rDNA gene (18S rDNA) (Acc. N.: AF190669). qPCR was performed in triplicates per sample using Biorad qPCR machine (Bio Molecular Systems, Upper Coomera, Australia). Briefly, each reaction was carried out in a final volume of 20 μL containing 1X TaqMan universal Master Mix (Applied Biosystems, Foster City, USA), 0.3 μM of each primer (PKDtaqf1: 5′–GCGAGATTTGTTGCATTTAAAAAG–3′ and PKDtaqr1: 5′– GCACATGCAGTGTCCAATCG–3′), 0.2 μM of the probe PKD (5′– CAAAATTGTGGAACCGTCCGACTACGA–3′), 1× of internal control Exo IPC Mix, 1× of IC DNA (TaqMan Univ. MMix w Exog IntPostC, Applied Biosystems), and 2 μL of template DNA. An initial denaturation step of 10 min at 95°C was followed by 45 cycles of 15 s at 95°C and 60 s at 60°C with fluorescence measurements after the latter step. Every qPCR run included a minimum of two non‐template controls (NTC; DNAse free water). A standard curve was generated for each run using five dilutions, ranging from 10^−5^ to 10^−8^ ng/μL of synthesized g‐Blocks. The regression coefficient of the standard curve had to be between −3.7 and −3.0, and the coefficient of variation between triplicates below 25% (Saura Martinez et al. 2025; Strepparava et al. 2018). If these criteria were not met, the sample was tested again. NTC within the qPCR never showed any amplification, while the internal control was always amplified showing no qPCR inhibition. Presence and abundance of the parasite (DNA copy number/μL) was determined. Number of positive fish and technical replicates were calculated per each sampling date. Brown trout and minnow were always handled separately.

qPCR positive samples were further investigated by conventional PCR according to (Kent et al. 1998), targeting a 298 bp region of the 18S rDNA gene of *T. bryosalmonae*. PCR was performed on an Eppendorf Mastercycler X50a (Eppendorf SE, Hamburg, Germany), using the forward primer PKX3F 5′–CTAAGTACATACTTCGGTAGA–3′ (10 μM) and reverse primer PKX4R 5′–CCGTTACAACCTTGTTAGGAA–3′. The PCR run included one negative control (NTC) and one positive control (containing original *T. bryosalmonae* DNA from positive brown trout kidneys). On a 1.8% agarose gel, 5 μL of the PCR product and buffer were run at 120 V for 25 min with a PowerPRO 300 (Thistle Scientific Ltd., Rugby, UK) and evaluated with an E‐BOX Gel Documentation Imaging System (Vilber, Collégien, France). When a band was present, 20 μL of the PCR product was then cleaned with a Wizard SV Gel and PCR Clean‐Up System (Promega Corporation, Fichtburg, Michigan, USA) according to manufacturer's instructions, and Sanger sequencing was carried out by Microsynth (Mycrosynth, Balgach, Switzerland).

To determine whether *T. bryosalmonae* was the only malacosporean parasite present in infected minnow or whether additional malacosporean species were detectable, PCR and sequencing were performed on selected kidney DNA samples using malacosporean‐specific primers described by Grabner and El‐Matbouli (2010a), with a modified forward primer to reduce species bias. BUTRA 2 (5–CAG CAG GCG CGC AAA TTA C–3′) and Budd‐r (5–CGA CCA AGC TCA AAC AAG TTT–3′) were used resulting in a 1100–1400 bp fragment of the 18S rDNA gene. The PCR was performed on the Eppendorf Mastercycler X50a with a total reaction volume of 22.5 μL, composed of 2.5 μL eDNA or DNA sample, 14.65 μL ddH_2_O, 2.25 μL PCR buffer (15 mM MgCl_2_), 0.99 μL of each forward (10 μM) and reverse primer (10 μM), 0.5 μL dNTPs (10 mM), 0.5 μL MgCl_2_ (25 mM), 0.12 μL HotStarTaq DNA polymerase. An initial denaturation step at 95°C for 15 min was followed by 40 three‐step amplification cycles, starting with a denaturation at 94°C for 45 s, followed by annealing at 58°C for 45 s and elongation at 72°C for 2.2 min, and a final elongation step of 10 min at 72°C. The PCR run included one negative control (NTC) and one positive control, same *T. bryosalmonae* DNA from positive brown trout kidneys as mentioned above. PCR products were purified using the NucleoSpinH ExtractII (Macherey Nagel, Düren, Germany). Sanger sequencing was carried out by Microsynth in both directions using the same primers as for the PCR.

Sequences obtained from Microsynth were processed with Mega11 Tamura et al. (2021). The forward sequence and the reverse complement of the corresponding reverse sequence were aligned, and the consensus sequence was manually corrected if needed. After primer sequence trimming the remaining Sanger sequences were aligned using MUSCLE (Edgar 2004) with default parameter setting and the resulting alignment was visualized in Jalview (Waterhouse et al. 2009). A nucleotide BLAST search (National Centre for Biotechnology Information (NCBI), Bethesda, Maryland, USA) was performed on all consensus sequences using blastn (megablast with default parameters) against the GenBank Nucleotide collection (nr/nt https://blast.ncbi.nlm.nih.gov) to identify the closest related sequences in the NCBI database.

### 2.8 Oxford Nanopore Technologies (ONT) Third—Generation Sequencing

Products of the prior 18S rDNA PCR with inconclusive or mixed Sanger sequencing results were processed by ONT deep amplicon sequencing in order to identify the potential individual species sequences. For the library preparation, the Nanopore Ligation sequencing amplicons—Native Barcoding Kit (SQK‐NBD114.24, ONT) were used. Purified PCR products were labelled with native barcodes. The protocol was carried out as described by the manufacturer, proceeding with the preparation of the DNA amplicon ends, barcoding, native adapter ligation and final library preparation (SQK‐NBD114.24, V NBA_9168_v114_revR_30jan2025). The final library contained 65 ng of library‐DNA in 12 μL, 37.5 μL of Sequencing buffer and 25.5 μL from Library Beads. The preparation and loading of the flow cell were carried out according to the manufacturer's instructions.

Sequencing was performed using the Flow Cell (FLO‐MIN114, R10 Version) and MinION device (Mk1D), in the software from ONT (MinKNOW) to collect raw data (.pod5 files). After sequencing, offline base‐calling (Super accurate model or SUP) was performed using the ONT's Dorado (1.2.0 version), with the command line dorado basecaller sup merged_output.pod5 – output‐dir to generate BAM output. Afterwards, the generated file was de‐multiplexed by using the demux function from Dorado (dorado demux – kit‐name SQK‐NBD114‐24 – output‐dir sup_demuxedbams *.bam – emit‐fastq) to generate FASTQ files from each barcode. To process these files, a specific pipeline (deposited at https://osf.io/dkbg5/overview?view_only=37bd16ecbe4c4eef86bbca47c01327c3) was developed using a dedicated bioinformatic environment (conda nanopore_env). Raw reads were first filtered and retained by quality and primer presence (any position and orientation). Afterwards, the retained reads were trimmed by removing barcodes and primers and a sequence length filter was applied (1100–1500 bp), followed by a dereplication and chimera removal step.

Operational Taxonomic Units (OTUs) were generated separately for each barcode by clustering sequences at different similarity thresholds. After analysing the OTUs generated with at least 50 reads (and considering quantity of OTUs and identity of the representative sequences), a similarity threshold of 99% was established. Using the individual barcode output results, a second part of the pipeline was conducted and referred to as OTU‐overall analysis, clustering all the filtered reads across all barcodes or samples and considering the number of reads included on each OTU and from each barcode. The output files (.tsv) generated after each processing step were imported into Microsoft Excel for a manual checking of each OTU sequence. Basecalling, demultiplexing and pipelines were run using command line tools in the Linux Terminal. The OTUs sequences were compared with reported sequences by BLASTn magablast (https://blast.ncbi.nlm.nih.gov).

### 2.9 Phylogenetic Analyses

The phylogenetic trees were constructed with most likelihood statistical analysis and a Bootstrap calculation based on 1000 replicates. 18S rDNA gene sequences were processed with MEGA11 with seven PKX‐amplicons (approx. 298 bp) or eleven BUTRA2/Buddr‐amplicon sequences (approx. 1300 bp), respectively. Sequences derived from brown trout kidney and eDNA samples were used as internal reference sequences to anchor *T. bryosalmonae* within the phylogenetic framework. Ingroup sequences were obtained of all five known species from the genus *Tetracapsuloides* (*T. bryosalmonae*) (KJ150286.1, AJ937884.1, MT002360.1, MG775220.1, KJ150284.1, MT002366, KF731712.1, U70623.1, KJ150285.1 and ON740974.1), *Tetracapsuloides* sp. 1 (KJ150278.1), *Tetracapsuloides* sp. 2 (KF731713.1), *Tetracapsuloides* sp. 3 (KF731715.1), *Tetracapsuloides* sp. 4 (KF731725.1), *Tetracapsuloides* sp. 5 (KF731729.1), sequences of five species of the malacosporean sister genus *Buddenbrockia* (*B. plumatellae*) (AY074915.1), *B. bryozoides* (MT002358.1), *Buddenbrockia* sp. 1 (KJ150261.1), *Buddenbrockia* sp. 2 (KF731700.1), *Buddenbrockia* sp. 3 (KF731708.1), *Buddenbrockia* sp. E (MT993755.1) and two not further classified malacosporean species *Malacosporea* sp. 1 (KJ150273.1) and *Malacosporea* sp. 2 (KJ150275.1). Three cnidarian sequences of *Hydra vulgaris* (XM_065807390.1), *Auralia aurita* (AY039208.1) and *Obelia geniculate* (KT722412.1) were set as outgroups.

### 2.10 Data Analysis and Statistics

Data was collected in a Microsoft Excel spreadsheet and imported in R (version 4.5.2, released 2025‐10‐31) (R Core Team 2026) using the package readxl 1.4.5 (Wickham and Bryan 2025). For data manipulation and transformation, the packages dplyr 1.2.0 (Wickham, François, et al. 2026) and tidyr 1.3.2 (Wickham et al. 2025) were used. Visualizations were created with the package ggplot2 4.0.2 (Wickham 2016; Wickham, Chang, and Henry 2026) and patchwork (Pedersen 2025). Survival analysis, including log‐rank test and Kaplan–Meier curves, was performed with packages survival 3.8–6 (Therneau 2026; Therneau and Grambsch 2000) and survminer 0.5.2 (Kassambara et al. 2026).

## 3 Results

### 3.1 Initial Fish Health Check

During the initial health check, histological examination revealed metacercariae of the trematode *Diplostomum phoxini* in the brains of three minnow. Five minnow showed a macroscopically distended abdomen with adult nematodes (*Philometra* sp.) free in the abdominal cavity. Both parasitic infections were considered potential additional stress factors but were not expected to influence the outcome of the experimental infection. Treatment of these parasites was not feasible within the framework of the experiment. Prior to experimental exposure, sudden increased mortality occurred in brown trout. Necropsy and histopathological examination revealed severe gas bubbles within gill capillaries, consistent with gas bubble disease caused by gas supersaturation. The cause of supersaturation could be resolved before the start of the exposure experiment. No further mortality attributed to gas bubble disease was observed after the start of the experiment. Neither minnow nor brown trout tested positive for *T. bryosalmonae* DNA.

### 3.2 Survival, Clinical Signs, and Histopathology

Minnow showed no mortality during the entire experimental period (Figure 1). In contrast, brown trout experienced high mortality, especially between 38 and 59 dpe. At 95 dpe, all brown trout died or were sampled, preventing further sampling. Log‐rank test showed a significant difference in survival between minnow and brown trout (Figure 1). An overview of fish mortalities during the experiment (including numbers and causes) is available in the open‐source OSF depository (data availability statement).

**FIGURE 1:**
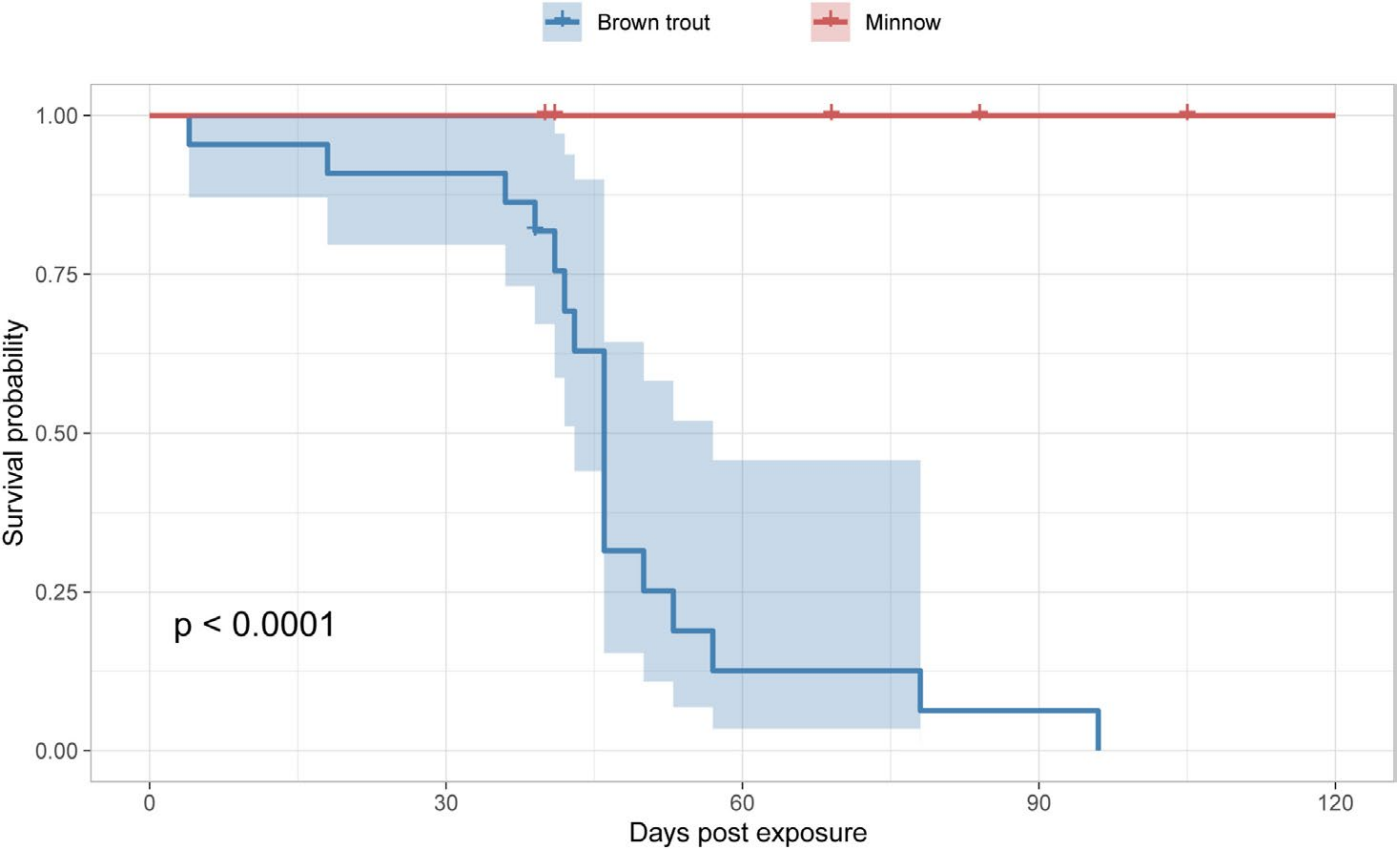
Kaplan–Meier survival curves of brown trout and minnow following infection. Survival probability of brown trout (*Salmo trutta fario*) and minnow ( *Phoxinus phoxinus* ) over time post exposure (days). Brown trout exhibit a marked decline in survival, whereas minnow show no mortality throughout the experimental period. Shaded areas represent 95% confidence intervals. Tick marks indicate predetermined sampling events (80 minnow and 5 brown trout), with the first sampling of minnow conducted over two consecutive days. Survival differed significantly between species (log‐rank test, *χ* ^2^ (1) = 186, *p* < 0.0001).

Minnow exhibited no clinical signs consistent with PKD, and their kidneys showed neither macroscopic proliferative changes (Figure 2A). In contrast, brown trout exhibited pronounced renal proliferation, particularly in the posterior region of the kidney (Figure 2B). Histologically, kidneys of minnows showed no PKD related lesions (Figure 2C), whereas brown trout exhibited a severe interstitial granulomatous nephritis, characterized by infiltration with mainly macrophages, accompanied by haemorrhage and interstitial necrosis. In addition, multiple interstitial *T. bryosalmonae*‐stages were observed (Figure 2D).

**FIGURE 2:**
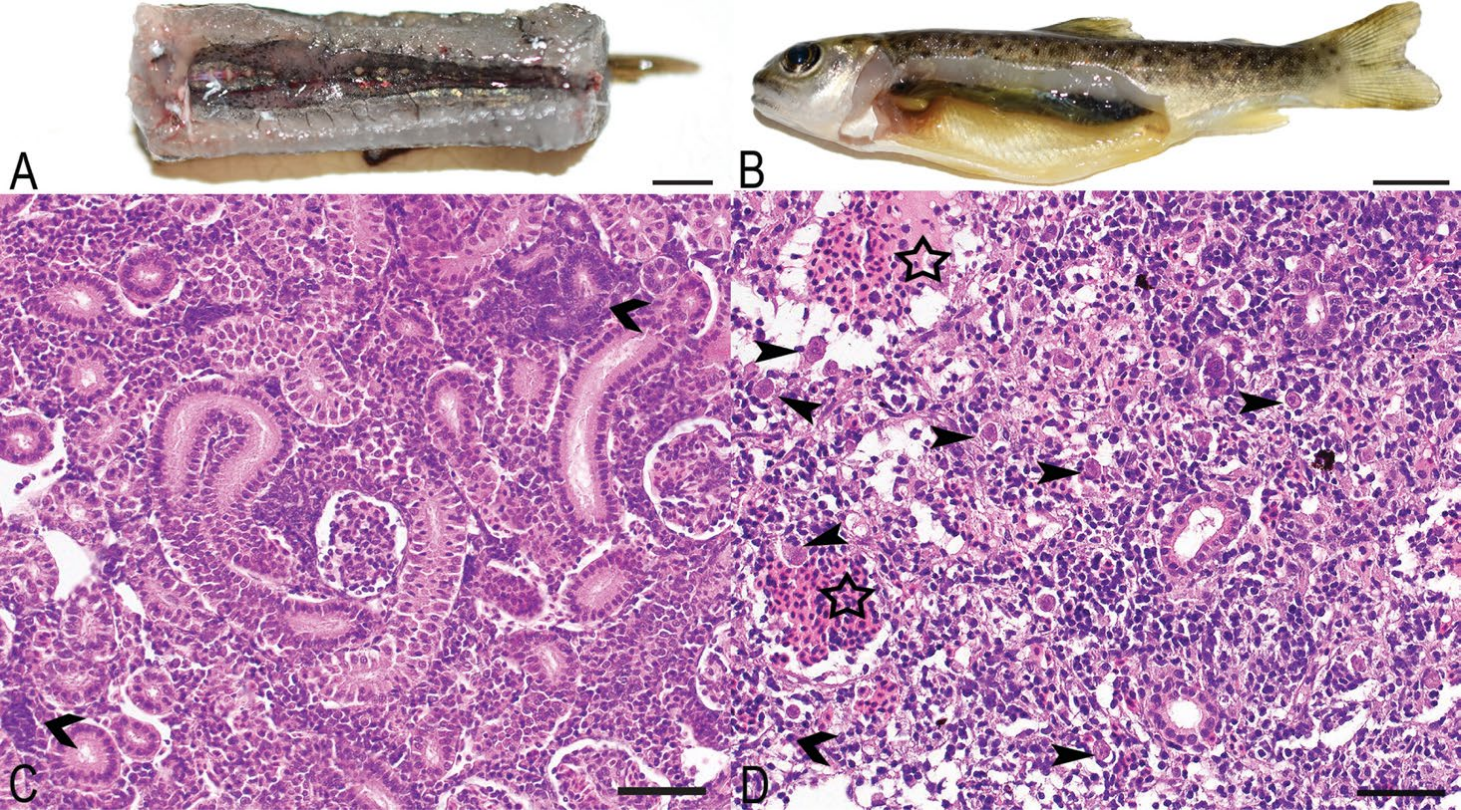
Macroscopic and histological appearance of kidney in brown trout and minnow. (A, C) Minnow ( *Phoxinus phoxinus* ); (B, D) Brown trout (*Salmo trutta fario*). Gross morphology of the kidney showing (A) no evident external abnormalities in the minnow kidney, ventral view, and (B) extensive swelling of the kidney in the posterior kidney region in ventrolateral view. (C, D) Histological sections of renal tissue (H&E stain). Minnow (C) show largely preserved renal architecture without pathology, mild tubuloneogenesis (open arrowheads). In brown trout (D), renal tissue exhibits marked pathology, including multifocal necrosis, haemorrhage and necrotizing vasculitis (open stars), with numerous interstitial developmental stages of *Tetracapsuloides bryosalmonae* (i.e., closed arrowheads). Scale bars: A = 0.5 cm, B = 1 cm; C, D = 50 μm.

### 3.3 PCR of Fish Kidney

One‐hundred and two fish kidneys (80 minnow and 22 brown trout) were examined by qPCR for *T. bryosalmonae* DNA. Five minnow kidneys (Minnow kidney (MK) 1–5, 6.25%) yielded positive qPCR signals. All of these positive results were obtained at the first sampling time point at 41 dpe. In three of the five positive animals (MK 3–5), only little amounts of parasite material were detected (1 out of 3 triplicates positive, with concentrations ranging from 0.36 to 2.03 copies/μL). One of the five animals (MK 2) tested positive in 2 out of 3 replicates (mean concentration 1.31 copies/μL), and one animal (MK 1) showed a mean concentration of 29.26 copies/μL (3 out of 3 replicates positive). All positive results were re‐tested for confirmation using two conventional PCR assays. The specific PKX PCR yielded one positive result (MK 1), and the non‐specific malacosporean PCR produced two positive results (MK 1 and MK 2, Table 1).

**TABLE 1:** Detection of malacosporean parasites across two minnow ( *Phoxinus phoxinus* ) kidneys, two brown trout (*Salmo trutta fario*) kidneys and nine environmental samples (eDNA) using *Tetracapsuloides bryosalmonae* specific qPCR and PCR assays targeting different amplicon lengths. Additionally, general malacosporean PCR with subsequent Sanger sequencing results (accession; identity).

Sample ID	Tank; Sampling point/days post exposure (dpe)	*Tetracapsuloides bryosalmonae* qPCR‐ (73 bp amplicon)	*Tetracapsuloides bryosalmonae* specific PKX PCR (298 bp amplicon)	General malacosporean (g.Mal.) PCR (1300–1400 bp amplicon)	Consensus length of g.Mal.‐PCR (bp)	Best BLASTn match of g. Mal.‐PCR (accession; identity)
Kidney samples						
MK 1^a^	Minnow Sampling 1	+	+	+	920	*Tetracapsuloides bryosalmonae* (KJ150286.1; 99.67%)
MK 2^a^	Minnow Sampling 1	+	−	+	No overlap	
BTK 1	Brown trout sampling 1	+	+	+	1272	*Tetracapsuloides bryosalmonae* (MT002366.1; 99.92%)
BTK 2	Brown trout sampling 1	+	+	+	1272	*Tetracapsuloides bryosalmonae* (MT002366.1; 99.92%)
eDNA samples						
eDNA M 9	Minnow; 53 dpe	+	+	+	1292	*Buddenbrockia bryozoides* (MT002365.1; 99.15%)
eDNA M 11^a^	Minnow; 53 dpe	+	+	+	1195	*Buddenbrockia bryozoides* (FJ939289.1; 99.49%)
eDNA M 13	Minnow; 53 dpe	+	−	+	1296	*Buddenbrockia bryozoides* (MT002358.1; 99.85%)
eDNA M 14	Minnow; 53 dpe	+	−	+	1142	*Buddenbrockia plumatellae* (KF731690.1; 100%)
eDNA M 15	Minnow; 56 dpe	+	−	+	1280	*Buddenbrockia plumatellae* (KF731682.1; 99.92%)
eDNA M 16	Minnow; 63 dpe	+	−	+	312	*Buddenbrockia plumatellae* (FJ939289.1; 100%)
eDNA M 19	Minnow; 66 dpe	+	−	+	No overlap	
eDNA BT 7	Brown trout; 49 dpe	+	+	+	1268	*Tetracapsuloides bryosalmonae* (FJ981823.1; 99.92%)
eDNA BT 8	Brown trout; 53 dpe	+	+	+	1275	*Tetracapsuloides bryosalmonae* (FJ981823.1; 99.84%)

*Note:* Positive amplification is indicated by (+) and absence of amplification by (−).

Abbreviations: BTK, brown trout kidney; eDNA BT, eDNA sample from brown trout aquarium; eDNA M, eDNA sample from minnow aquarium; MK, minnow kidney.

^a^ Denote samples which were further investigated using Oxford Nanopore Technology (ONT) deep amplicon sequencing. Minnow kidney and eDNA samples that were not confirmed by either conventional PCR assay (3 minnow kidney samples, 12 minnow eDNA samples) were excluded from the table.

In brown trout, kidney samples (Brown trout kidney, BTK) consistently tested positive (3 out of 3 triplicates) for parasite DNA across all sampling points. The parasite DNA concentrations ranged from 4818.77 to 4,642,805.03 copies/μL. *T. bryosalmonae* DNA was confirmed in two samples (BTK 1 and 2) by PKX and malacosporean PCR assays (Table 1).

### 3.4 eDNA

eDNA from infected fish was consistently detected in tank water from both host species, but temporal dynamics and concentrations differed markedly between minnow and brown trout. For minnow, eDNA concentrations rose from very low concentrations in the early 27 to 35 dpe (0.01–0.63 copies/μL) to low but consistently detectable levels (2.56–51.7 copies/μL) during the mid‐experimental period (49–91 dpe) (Figure 3A). Limit of detection (LOD) was recently calculated by Stelzer et al. (2024) at 3.59 copies/μL using the qPCR assay by Bettge, Segner, et al. (2009). Filter replicates were inconsistently positive, with frequent alternation between positive and negative samples across tanks and sampling points.

**FIGURE 3:**
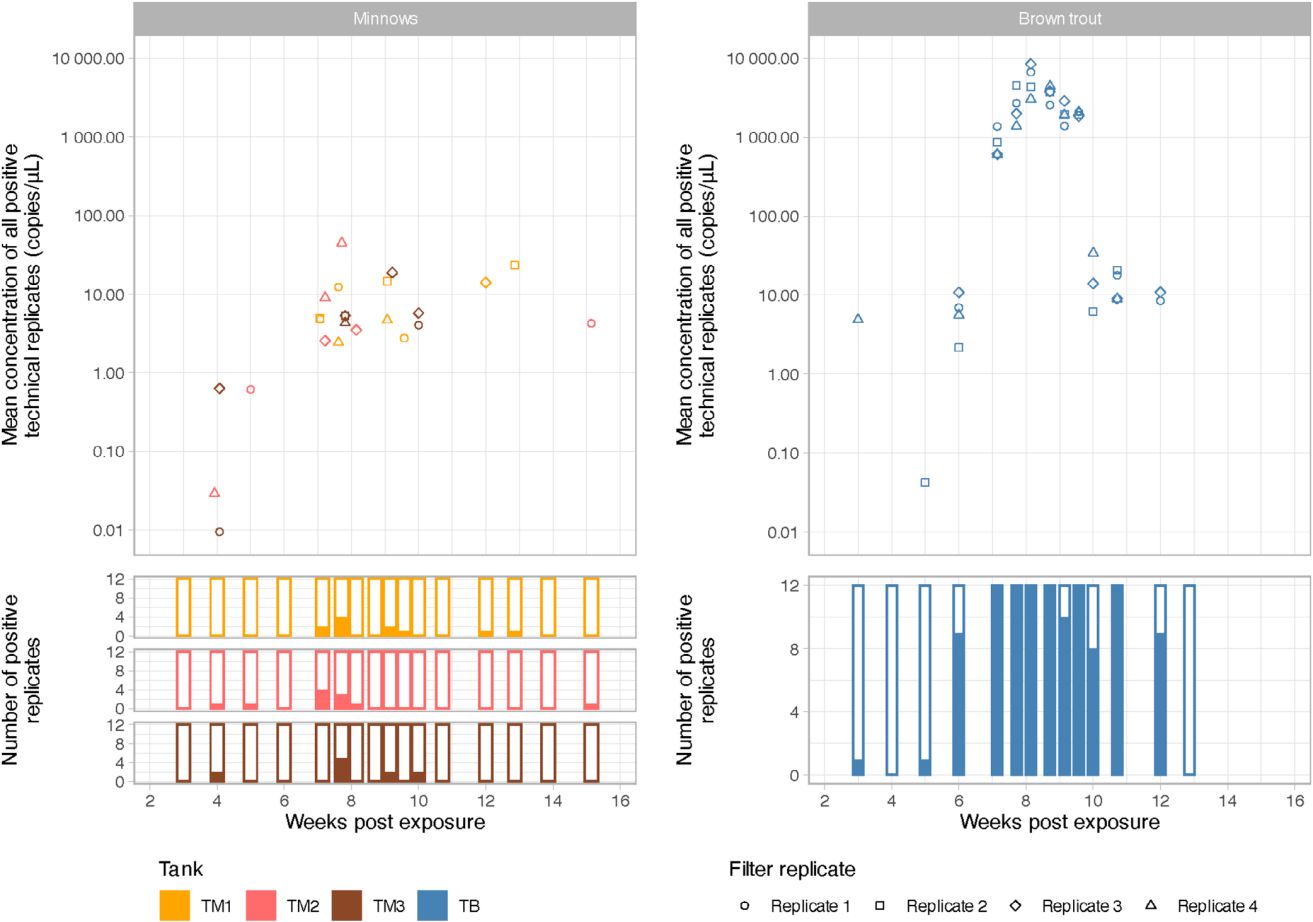
Temporal dynamics of *Tetracapsuloides bryosalmonae*‐positive eDNA detections in minnow and brown trout over the experimental sampling period. (A) Minnow ( *Phoxinus phoxinus* ) and (B) Brown trout (*Salmo trutta fario*). The upper plots display the mean concentration of all positive technical replicates (copies/μL) over weeks post exposure on a logarithmic scale, with symbols indicating individual Sterivex‐filter replicates (○, □, ◇, △) and colours denoting tank (TM1‐3, TB). The lower panels show the cumulative number of technical replicates per tank and sampling point over time, while filled bars represent the number of qPCR positive technical replicates. Unexposed minnow tank (not shown) filter replicates showed no amplification.

In contrast, brown trout produced substantially stronger eDNA signals. Mean parasite DNA concentrations rapidly increased after infection with first detection at 21 dpe and peaked between 58 and 65 dpe, reaching concentrations of several hundred to several thousand copies/μL and forming a distinct high‐shedding phase in the middle of the experiment (Figure 3B). During this period, nearly all filter replicates from all tanks tested positive. At later sampling points, eDNA concentrations and the number of positive replicates declined, although signals generally remained stronger and more persistent than in minnow. Positive eDNA results persisted until 83 dpe (Figure 3B).

### 3.5 Sanger Sequencing, ONT Deep Amplicon Sequencing, and Phylogenetic Tree

Five kidney samples (MK 1–5) from minnow tested positive by qPCR. Signals were generally weak except in one case (MK 1) with a strong consistent signal. All positive kidney samples originated from the first sampling time point (one from TM1, two from TM2, and two from TM3). These five samples were retested with conventional PCR PKX‐specific primers, and one kidney (MK 1) was confirmed for *T. bryosalmonae*. The five qPCR positive samples were subsequently retested for a longer amplicon sequence with conventional malacosporean primers. The same sample (MK 1) and one additional sample (MK 2) yielded amplification with Sanger sequencing confirming *T. bryosalmonae* and *Tetracapsuloides* sp. 3, respectively. The remaining samples did not produce consistent amplification with either primer set (Table 1).

A total of 19 eDNA samples (between days 28 and 91) originating from the minnow tanks yielded positive qPCR signals (eDNA M 1–19). Of these, two samples were confirmed using subsequent conventional PKX‐specific assay (eDNA 9 and 11). Seven qPCR positive eDNA samples (eDNA M 9, 11, 13, 14, 15, 16, 19) showed positive amplification with general malacosporean primers. The general malacosporean amplicon sequences of eDNA M 9, 11, and eDNA M 13–16 were blasted for *Buddenbrockia bryozoides* and *B*. *plumatellae* (Table 1) eDNA M 19 yielded a visible band; however, no successful overlap was obtained from the Sanger sequencing reads. The remaining qPCR‐positive eDNA samples were not confirmed by either PKX‐specific or general malacosporean primers.

Two kidney (BTK 1 and BTK 2) and two eDNA (eDNA BT 7 and 8) samples from brown trout were included as positive controls and as reference material for phylogenetic comparison. Both brown trout kidney samples and eDNA samples showed consistent positive amplification across all assays (qPCR, PKX‐specific PCR, and general malacosporean PCR), confirming the presence of *T. bryosalmonae* (99.84%–99.92% identity).

Two kidney samples (MK 1 and 2) along with the eDNA sample 11 were selected for high‐resolution deep amplicon sequencing using ONT. General malacosporean 18S rDNA amplicons were processed for potential identifying underlying mixed infection. ONT sequencing could fully corroborate Sanger sequencing results and excluded an underlying sequence heterogeneity in all three samples. In particular, sample MK 1 yielded a total of 3206 reads of *T. bryosalmonae* without a detectable co‐infection (Table 2).

**TABLE 2:** Summary of Oxford Nanopore Technologies (ONT) sequencing results for selected kidney (MK 1, MK 2) and environmental (eDNA M 11) samples. Reported are sequence length, total read counts, and best BLASTn matches including GenBank accession numbers and percentage identity.

Sample ID	Length (bp)	Total reads	Best BLASTn match (accession; identity)
MK 1	1324	3206	*Tetracapsuloides bryosalmonae* (FJ981823.1; 99.77%)
MK 2	1328	5742	*Tetracapsuloides* sp. 3 (KF731717.1; 99.62%)
eDNA M 11	1344	10,935	*Buddenbrockia bryozoides* (MT002358.1; 99.93%)

Phylogenetic analyses for constructing the tree were exclusively based on sequences generated from the conventional PKX‐PCR assay (200 bp). Sequences obtained from one minnow kidney sample (MK 1), two brown trout kidney samples (BTK 1 and 2), as well as eDNA samples from minnow (eDNA M 9 and 11) and brown trout (eDNA BT 7 and 8) were aligned with ten reference *T. bryosalmonae* sequences retrieved from NCBI. The alignment was analysed using a phylogenetic tree, which revealed a clear and distinct clustering of all experimentally derived PKX sequences within the *T. bryosalmonae* clade (Figure 4).

**FIGURE 4:**
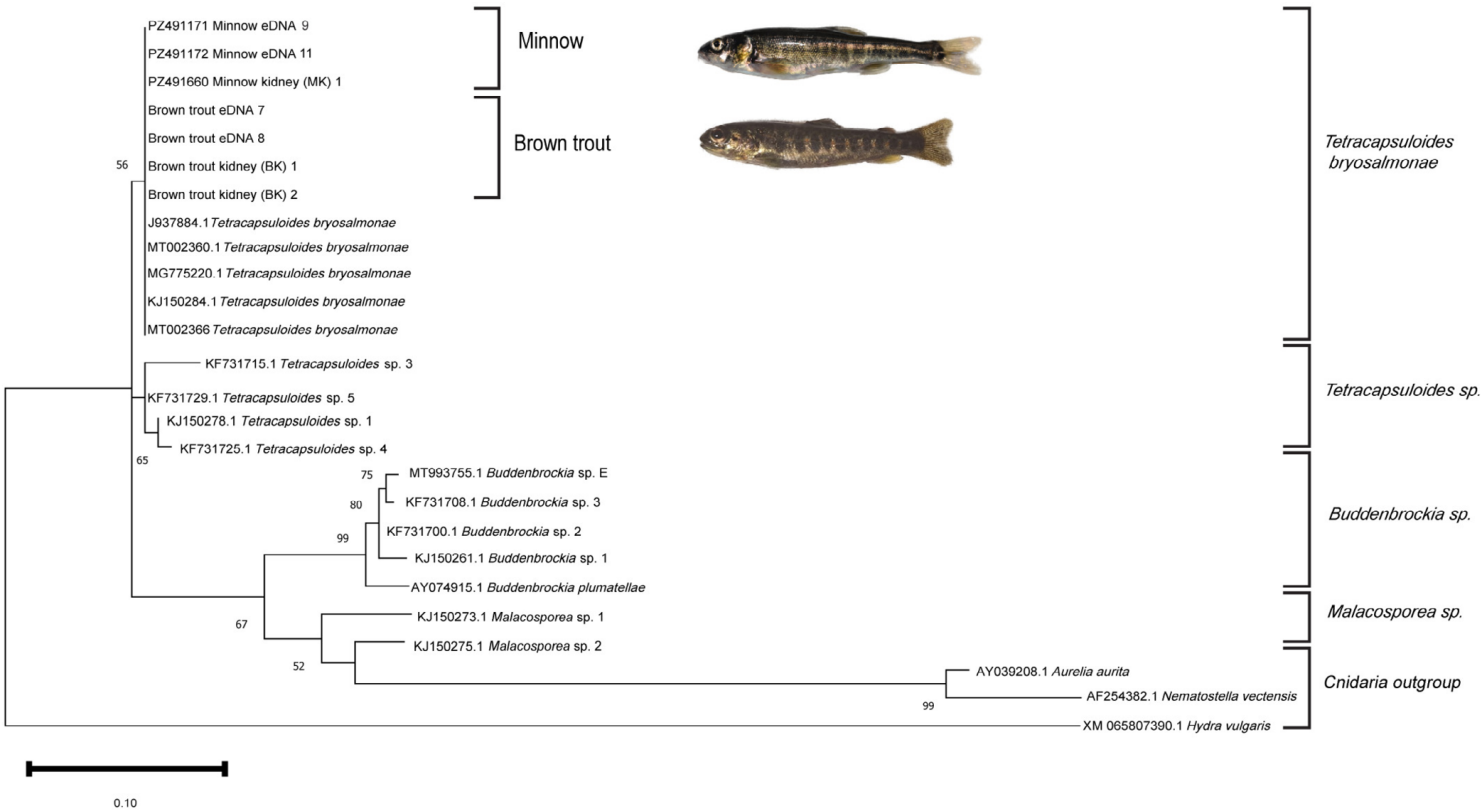
Phylogenetic relationships of *Tetracapsuloides bryosalmonae* sequences obtained from minnow (MK 1, eDNA M 9 and 11) and brown trout samples (BK 1/2 and eDNA BT 7/8) based on PKX‐PCR partial 18S rDNA gene sequences. The analysis included nucleotide sequences of partial sequence of 18S rDNA gene of *Tetracapsuloides* and *Buddenbrockia* species. The phylogenetic tree was constructed using the Maximum Likelihood method based on the Tamura–Nei model. Bootstrap support values (% of 1000 replicates) are indicated at the nodes. Branch lengths are drawn to scale and represent the number of substitutions per site. Three cnidaria relatives were used as outgroups. All analyses were conducted using MEGA11.

Overall, phylogenetic results are consistent with a close relationship to other *Tetracapsuloides* species (with exception of *Tetracapsuloides* sp. 2, for which no comparable PKX sequence data were available) and form a sister clade to *Buddenbrockia* species.

## 4 Discussion

**First confirmation of an additional intermediate host species in the *T. bryosalmonae* life cycle**. Our infection experiment demonstrated that minnow can sustain *T. bryosalmonae* infections under controlled laboratory conditions and may release parasite DNA, as indicated by positive eDNA qPCR and conventional PKX‐PCR results, confirmed by Sanger sequencing. However, this was not confirmed by ONT deep amplicon sequencing. Overall, minnows exhibited lower infection competence compared to classical salmonid hosts such as brown trout. However, their potentially lifelong low‐level excretion, as *T. bryosalmonae* infection did neither induce clinical signs nor mortality, may compensate for reduced shedding intensity and could nevertheless make them relevant contributors to environmental parasite reservoirs. Especially, as minnows are used in some countries as live bait (Mulligan 2023), they could facilitate the spread of *T. bryosalmonae* and therefore PKD into previously uninfected river systems, posing a significant threat to vulnerable salmonid populations already stressed by warming temperatures and habitat degradation (Ros et al. 2022). Understanding the infection competence of minnows is therefore not only critical for clarifying the parasite's host range but also essential for informing biosecurity measures, fisheries regulations, and conservation strategies aimed at protecting sensitive salmonid stocks.

Myxozoans are known to infect atypical hosts without supporting full spore development (Bartošová‐Sojková et al. 2014; Holzer et al. 2018; Shivam et al. 2023). In line with our results, we reveal that minnow can harbour *T. bryosalmonae* and possibly sustain genuine *T. bryosalmonae* infections. This is evidenced by qPCR positivity, sequencing‐confirmed PCR products, and long‐read deep amplicon sequencing that unambiguously identified *T. bryosalmonae* DNA from minnow kidney, ruling out closely related *Tetracapsuloides* species and overcoming short‐amplicon limitations (Bettge, Segner, et al. 2009; Kent et al. 1998). The parasite was also traced to eDNA shed by minnow via qPCR and PKX‐specific PCR (Kent et al. 1998; Stelzer et al. 2024). Yet, eDNA proved elusive for broader confirmation; neither malacosporean‐specific PCR nor long‐read sequencing succeeded, likely due to admixture with other malacosporean DNA in higher concentration like *Buddenbrockia* sp. Long‐read eDNA sequencing instead identified the sister malacosporean *B. plumatellae*, a low‐pathogenic kidney invader previously shown in minnow (Grabner and El‐Matbouli 2010a). Sharing the same bryozoan host *F. sultana* with *T. bryosalmonae*, it sets up a plausible co‐infection scenario via wild colonies (Canning et al. 2002). Our eDNA results indicate that *Buddenbrockia* may outcompete *T. bryosalmonae* in dual infections. While *Buddenbrockia* was unquantified in bryozoans, sequencing affirmed both parasites, showing a plausible co‐infection in minnow kidneys and eDNA.

**Absence of PKD specific signs.** The absence of PKD‐related clinical signs in minnow, such as anaemic gills, abdominal swelling, or kidney and spleen enlargement (Hedrick et al. 1993), suggests fundamental host‐specific differences in parasite–host interaction. In salmonids, PKD pathology is driven not only by the presence of the parasite but by extensive proliferation of extrasporogonic stages and the resulting dysregulation of the immune system, which culminates in severe lymphoid proliferation and granulomatous and necrotising nephritis (Abos et al. 2018; Bailey et al. 2019; Kumar et al. 2015). Kidney of cyprinids (where minnow belong to) may lack the cellular receptors, immunological architecture, or signalling environment necessary to support this proliferation, preventing the parasite from establishing the pathogenic stages seen in salmonids (Kumar et al. 2015; Coxon et al. in preparation). Such interspecific differences in immunological response and tolerance are well documented even among salmonids, for example, between brown trout, a true intermediate host, and rainbow trout, a dead‐end host. Brown and rainbow trout display distinct B‐ and T‐cell responses and divergent evolutionary adaptations (Bailey et al. 2019; Chilmonczyk et al. 2002; Holland et al. 2003; Ord et al. 2026). It is therefore plausible that minnow have developed effective mechanisms to limit parasite development before tissue‐damaging stages arise. Consistent with this, histological examination in our study revealed no PKD‐associated pathology in any minnow kidney section. Rapid innate immune clearance, as reported for other myxozoans (Alama‐Bermejo et al. 2019; Sitjà‐Bobadilla 2008), could prevent establishment of early tissue stages. This corresponds to the fact that many myxozoan infections are innocuous or inapparent (Hartikainen et al. 2016). The combination of negative histology and positive kidney PCR results implies that these species are experimentally infected yet exhibit a high degree of tolerance, potentially functioning as well‐adapted intermediate or reservoir hosts. However, an alternative explanation is that they act as dead‐end hosts incapable of supporting development and excretion of infectious spores. In our study, *T. bryosalmonae* DNA was detected in minnow eDNA solely by qPCR but not confirmed by long‐read sequencing. Thus, it has not been conclusively demonstrated that the parasite is actively shed by minnow. Further evidence, such as successful reinfection of bryozoans ( *F. sultana* ), would be required to definitively establish minnow as transmission vectors in the *T. bryosalmonae* life cycle.

**Economic and ecological value of minnow in comparison to brown trout.** The detection of *T. bryosalmonae* in minnow raises important questions about why this host–parasite association has not been observed before. Several non‐mutually exclusive explanations are plausible. Firstly, the low pathogenicity of *T. bryosalmonae* in this host may result in subclinical or asymptomatic infections that previously escaped attention. Moreover, the minnow is a small, abundant species that has historically received far less attention than trout (Frost 1943; Scharnweber 2020). Minnow possess little to no economic value and were historically used as live‐bait fish in some countries, contributing to their widespread translocation and introduction into new regions and ecosystems (Gozlan et al. 2010; Scharnweber 2020). Another possibility is that earlier sampling and diagnostic approaches lacked sufficient sensitivity. Even in salmonids, *T. bryosalmonae* can persist chronically in surviving fish. For instance, in brown trout the parasite remains PCR‐detectable up to 5 years post‐exposure, even in the absence of active disease, and such chronically infected individuals can still shed infective stages to bryozoans (Soliman et al. 2018). Comparable latent low‐level infections in minnow would be extremely difficult to detect. Historically, detection of *T. bryosalmonae* relied mainly on histology, immunohistochemistry (IHC), or conventional PCR, but studies have demonstrated the superior sensitivity of molecular methods, particularly qPCR (Seidlova et al. 2021; Bettge, Segner, et al. 2009). Seidlova et al. (2021) found that IHC detected only 30% of infected salmonids, whereas conventional PCR and qPCR detected 65.4% and 81.9% of cases, respectively. As diagnostic tools have advanced, infections that were once undetectable can now be reliably identified, including in previously overlooked, non‐traditional hosts such as minnow.

**Minnow as pathogen carrier**. The minnow co‐occurs with salmonids in the field and may therefore play an important role as reservoirs for pathogens such as *T. bryosalmonae*. Unlike salmonids, these species can tolerate warmer, less turbulent waters and exhibit relatively broad temperature tolerances (Frenette et al. 2019). In contrast, salmonids are stenothermic and require cold, well‐aerated habitats, particularly during early life stages (Elliott and Elliott 2010; Rubin et al. 2019). With climate change, temperatures in salmonid spawning habitats are expected to increase (Ros et al. 2022), therefore, cohabitation between salmonids and non‐salmonid fishes, such as minnow, is likely to become more common. If these non‐salmonid species serve as reservoirs or intermediate hosts of *T. bryosalmonae*, the parasite may spread into previously unaffected habitats, placing YOY brown trout at elevated risk of infection. Such a scenario would necessitate targeted management interventions, including the preservation and restoration of cold, turbulent freshwater habitats (Ros et al. 2022), as well as selective restocking of salmonids into habitats free of the implicated non‐salmonid species.

## 5 Conclusions

Our study provides the first comprehensive molecular and experimental evidence that minnow can acquire and harbour *T. bryosalmonae* infections. By demonstrating the presence of DNA in renal tissue and potential subsequent parasite shedding into the water column, our findings suggest that the host range could be broader than currently recognised and that alternative fish hosts may play a more important role in the transmission ecology of PKD than previously estimated.

However, additional life‐cycle closure experiments are needed to determine whether minnows contribute to onward transmission or function only as transient carriers. In particular, exposing naïve *F. sultana* colonies to water or excretions from experimentally infected minnows will be essential to assess whether viable malacospores can develop within the bryozoan host. Because peak parasite shedding in minnows occurs around 7 wpe, this period provides a targeted window for such trials.

## Author Contributions

**Heike Schmidt‐Posthaus:** conceptualization, funding acquisition, writing – original draft, writing – review and editing, validation, project administration, data curation, supervision, resources, formal analysis. **Céline Möckli:** investigation, writing – original draft, methodology, validation, visualization, writing – review and editing, formal analysis, data curation. **Nicolas Diserens:** writing – review and editing, conceptualization, formal analysis, project administration, supervision. **Gary Delalay:** formal analysis, visualization, writing – review and editing. **Gastón Moré:** writing – review and editing, methodology, data curation.

## Funding

The project was supported by the Federal Office for the Environment (06.0121.PZ/504C1E164) and Swisslos‐Fonds canton Aargau (2023.REF 5979).

## Conflicts of Interest

The authors declare no conflicts of interest.

## Data Availability

Novel Sequences of minnows were deposited in the GenBank database. PKX sequences were registered under accession numbers PZ491660 (MK 1), PZ491171 (eDNA M 9), and PZ491172 (eDNA M 11). Malacosporean sequences were deposited under accession numbers PZ499066 (MK 1) and PZ499067 (MK 2). All original data are available in OSF open data depository (https://osf.io/dkbg5/overview?view_only=37bd16ecbe4c4eef86bbca47c01327c3).
